# Molecular docking based virtual screening of the breast cancer target NUDT5

**DOI:** 10.6026/97320630015784

**Published:** 2019-12-05

**Authors:** Razia Sultana, Monjia Islam, Md.Azizul Haque, Fatematuz Zuhura Evamoni, Zahid Mohammad Imran, Jabunnesa Khanom, Md.Adnan Munim

**Affiliations:** 1 Department of Biotechnology and Genetic Engineering, Faculty of Science, Noakhali Science and Technology University, Noakhali-3814; 2 Department of Applied Chemistry and Chemical Engineering, Faculty of Engineering and Technology, Noakhali Science and Technology University, Noakhali-3814; a Equal contribution

**Keywords:** Breast cancer, NUDT5 protein, Homology modelling, Molecular docking

## Abstract

Breast cancer affects one in eight women in Bangladesh and is the most common cancer among women in South Asia next to skin cancer. NUDT5 are nucleotide-metabolizing enzymes (NUDIX hydrolases) linked with the ADP ribose and 8-oxo-guanine metabolism. It is known to be associated with the hormone dependent gene regulation and proliferation in breast cancer cells. It blocks progestin-dependent, PAR-derived nuclear ATP synthesis and subsequent chromatin remodeling, gene regulation and proliferation in this context. We describe the structure based binding features of a lead compound (7-[[5-(3, 4-dichlorophenyl)-1,3,4-oxadiazol-2-yl]methyl]-1,3-dimethyl-8piperazin-1yl-purine-2,6-dione-C_20_H_20_C_l2_N_8_O_3_) with NUDT5 for further in vitro and in vivo validation. It is a promising inhibitor for blocking NUDT5 activity. Thus, structure based virtual screening is used to identify a potential therapeutic inhibitor for NUDT5.

## Background

Breast cancer is the most lethal disease in women and is the second leading cause of cancer-related deaths in the United States [1] with symptoms such as a lump in the breast, a change in breast shape, dimpling of the skin and fluid coming from the nipple, a newly-inverted nipple, or a red or scaly patch of skin [4] depending on cancer type/stage and patient's age [2]. Survival rates in the developed world are high [3], with between 80 and 90% in England and the United States alive for at least 5 years[5,6].Worldwide, breast cancer is the leading type of cancer in women, accounting for 25% of all cases, in 2018 with 2 million new cases and 627,000 deaths [6]. The enzyme NUDT5 (nucleotide diphosphate hydrolase type 5) catalyzes the ADP (Adenosine diphosphate) ribose derived from hydrolysis of poly (ADP-Ribose) and pyrophosphate (PPi) are converted to ATP. It is known that NUDT5 is an upstream regulator of tumor drivers and are a biomarker for cancer stratification, as well as a target for drug discovery towards the treatment of aggressive cancer types and metastasis [7]. It is also known that NUDT5 protein plays noteworthy roles in regulating the G1-S transition in mammalian [9]. It is also known that NUDT5 (also referred as NUDIX5) has been linked to hormone dependent gene regulation and proliferation in breast cancer cells. The NUDIX hydrolases are a core family of nucleotide metabolizing enzymes that have critical roles in health and disease [11].Thus, NUDT5 is an attractive target for drug design against breast cancer.

**Figure 1:**
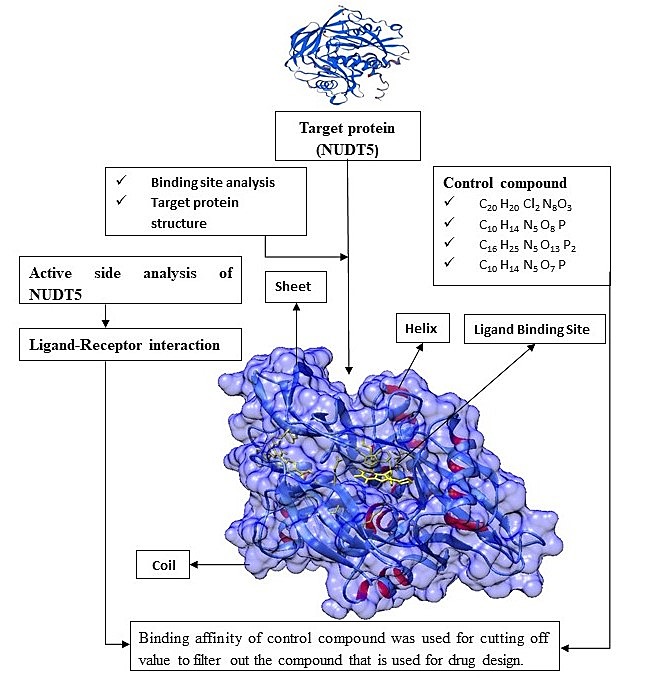
A representation of the enzyme-coupled malachite green assay (MG assay).ADPR is hydrolyzed to AMP and R5P by NUDT5,and then R5P is converted to free inorganic phosphate detected by the malachite green reagent.

The use of structure-based virtual screening for finding effective compounds for designing a drug against breast cancer is getting momentum in the last decade [10]. We used the structure NUDT5 (PDB ID: 5NWH) from protein data bank [12] for this study. We used protein-ligand complexes (PDB ID: 5NWH, 3BM4, 2DSD, 3L85) of NUDT5 from protein data bank (PDB). Two predominant substrates have been identified for NUDT5: 8 oxo -dGDP and adenosine 5' diphosphate[15-19] and these are important signally molecules [20-23]. It is further known that NUDT5 hydrolyzes 8-oxo-dGDP under basic conditions (pH ≈ 10) [15] as shown in Figure 1. Moreover, NUDT5 links with several critical events of cell progressing in relation to breast cells is known [23-24]. Therefore, it is of interest to use high throughput screening strategy to identify the small molecule inhibitor to NUDT5 using structure based drug design and molecular docking.

## Methodology

### NUDT5 protein model as target:

A reasonable protein model [27] of NUDT5 was developed using the tools Easy MODELLER ™ and SWISS-PROT.

### Active side prediction in NUDT5:

The residue around active site of the protein was predicted by using CASTp [28].The X-ray diffraction structure of the breast cancer regulator NUDT5 (PDB Code: 5NWH) in complex with twenty carbon compound 7-[[5-(3, 4-dichlorophenyl)-1,3,4-oxadiazol-2-yl]methyl]-1,3-dimethyl-8-piperazin-1-yl-purine-2,6-dione-C_20_ H_20_ Cl_2_ N_8_O_3_ resolved at 2.6 Å resolution were used as the basis of the docking experiments.

### Ligand compounds:

Four different ligands sourced from PDB (PDB ID: 5NWH, 3BM4, 2DSD, 3L85) used in this study (Table 1) are given below:

[1] 7-[[5-(3,4-dichlorophenyl)-1,3,4-oxadiazol-2-yl]methyl]-1,3-dimethyl-8-piperazin-1-yl-purine-2,6-dione-C_20_ H_20_ Cl_2_ N_8_ O_3_ [2] 8-oxo-2'-deoxy-guanosine-5'-monophosphate-C_10_ H_14_ N_5_ O_8_ P [3] alpha-beta methylene ADP-ribose-C_16_ H_25_ N_5_ O_13_ P_2_ [4] adenosine monophosphate-C_10_ H_14_ N_5_ O_7_ P 

### Docking validation:

The docking of the ligand 7-[[5-(3, 4-dichlorophenyl)-1,3,4-oxadiazol-2-yl]methyl]-1,3-dimethyl-8-piperazin-1-yl-purine-2,6-dione with theNUDT5 protein was done as described elsewhere [29] and their interaction featured were calculated using PyMOL [30] to assess docking efficiency.

### Structure based virtual screening:

The structural-based virtual screening (SBVS) were completed using VSDK (Virtual screening by docking) as described elsewhere [31-32]. VSDK were chosen mainly because (a) it is freely available (b) easy to operate and (c) it has an ability to take advantage of multiple core processors as well as have much more efficient search of the potential energy surface. Virtual screening was performed using PyRx AutoDock 4.

### Receptor-Ligand interaction:

Ligand-receptor interaction is illustrated using Discovery studio.

### Force field used in this study:

The default Force field in the Chimera software is used where required.

### Docking:

**Figure 2:**
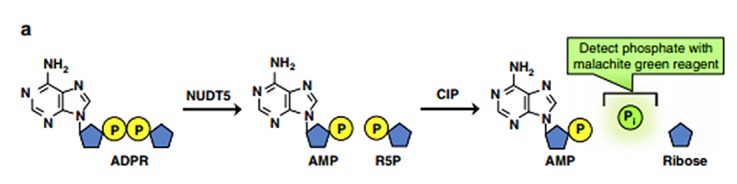
Workflow used in this study

Four ligands after applying force field were converted to Auto Dock ligand (pdbqt) and added them to the PyRx for docking with NUDT5 using the Vina Wizard. The interactions were analyzed usingPymol(http://pymol.sourceforge.net/) and Discovery Studio as described in Figure 2.

## Results and Discussion:

**Figure 3:**
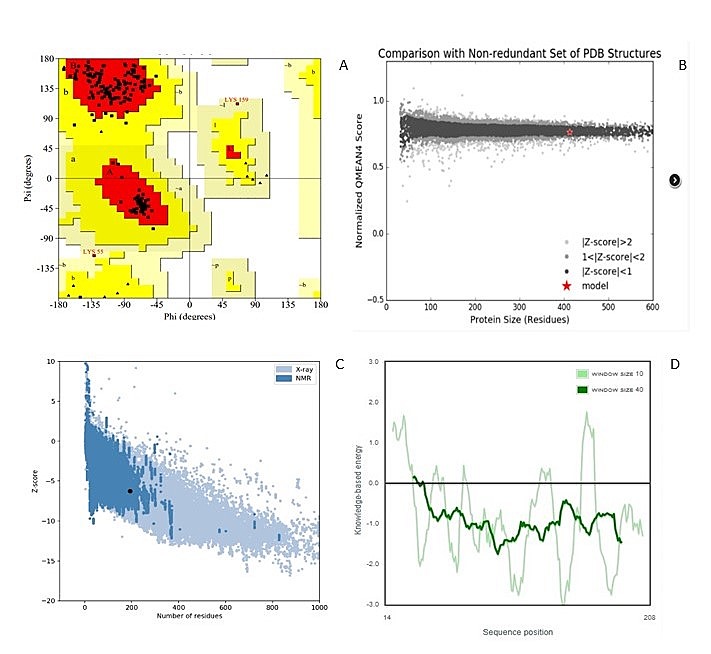
(A) The Ramachandran plotof NUDT5.(B) Qmean based structure validation, which compares the structure to a non-redundant set of PBDs of similar size. The NUDT5 structure, indicated using a red star, lies within the range of scores of similar size structures, indicating its good quality.(C)ProSa Z-score plot of the NUDT5 (D)The local quality of the model is shown in a plot of energy as a function of amino acid sequence position.

**Figure 5:**
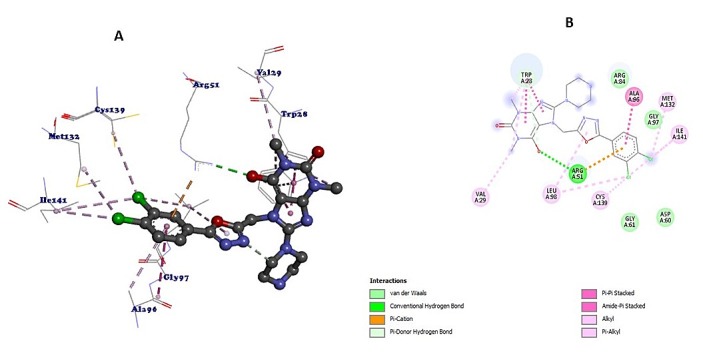
(A) Ligand interaction with the active site of the NUDT5.(B) 2D structure of ligand-receptor interaction

It is of interest to design inhibitors for the breast cancer target NUDT5 using molecular docking based virtual screening followed by molecular docking. A molecular model of NUDT5 was developed using Easy MODELLER using known templates with PDB IDs 5NQR, 3BM4and 3ACA and validated using standard procedures as shown in Figure 3. Potential binding sites were searched using the CASTp server [28]. The aminoacid residues involved in binding pockets are with ligand molecule are thus predicted. Nine possible binding residues such as ILE141, TRP28, ARG51, ALA96, VAL29, GLY97, LEU98, MET132, and CYS139were found to be involved in interaction with lead inhibitors. Thus, all of these were confirmed as important residues and used to create the grid for docking. The binding pocket has a volume of 392.039 Å and surface area of 555.407 as calculated by CASTp and shown inFigure 4.The interaction by the analysis of ligand-protein (NUDT5) usingDiscovery Studio is shown in Figure 5. Features such as hydrophobicity, H-Bond, Ionization, Aromatic compound and charges were calculated.PDB IDs 5NQR, 3BM4and 3ACA and validated using standard procedures as shown in Figure 3. Potential binding sites were searched using the CASTp server [28]. The aminoacid residues involved in binding pockets are with ligand molecule are thus predicted. Nine possible binding residues such as ILE141, TRP28, ARG51, ALA96, VAL29, GLY97, LEU98, MET132, and CYS139were found to be involved in interaction with lead inhibitors. Thus, all of these were confirmed as important residues and used to create the grid for docking. The binding pocket has a volume of 392.039 Å and surface area of 555.407 as calculated by CASTp and shown inFigure 4.The interaction by the analysis of ligand-protein (NUDT5) using Discovery Studio is shown in Figure 5. Features such as hydrophobicity, H-Bond, Ionization, Aromatic compound and charges were calculated.

A large number of docking program and search algorithms have been published during last 20 years. It is thus essential to conduct validation before use of these programs. Redocking could serve as validation procedure to determine whether the molecular docking algorithm is able to recover the crystallographic position using computer simulation [29]. In this study, docked ligand pose were compared to the intact ligand pose as measured using PYMOL. The interaction energies and properties were computed for the docked 7-[[5-(3,4-dichlorophenyl)-1,3,4-oxadiazol-2-yl]methyl]-1,3-dimethyl-8-piperazin-1-yl-purine-2,6-dione,8-oxo-2-deoxy-guanosine-5'-monophosphate, alpha beta methylene ADP ribose, Adenosine mono-phosphate using PyRxAutodock.2,6-dione exerted a highest binding free energy of-9.2 Kcal/mol and formed a single hydrogen bond with ARG51. All control compounds bind deeply into the binding site and show same overall conformation. Based on the docking result 7-[[5-(3, 4-dichlorophenyl)-1,3,4-oxadiazol-2-yl]methyl]-1, 3-dimethyl-8-piperazin-1-yl-purine-2,6-dione were used as positive control. Moreover, the docking studies provide useful insight into the mechanism of reference inhibitor binding to the active site.The Protein-ligand interaction plays a significant role in structure based drug designing. The best confirmation shows that the free energies of binding (ΔGbind kcal/mol) for the four ligands were -9.2 kcal/mol, -7.4 kcal/mol and -6.5 kcal/mol and -6.4 kcal/mol.

**Figure 6:**
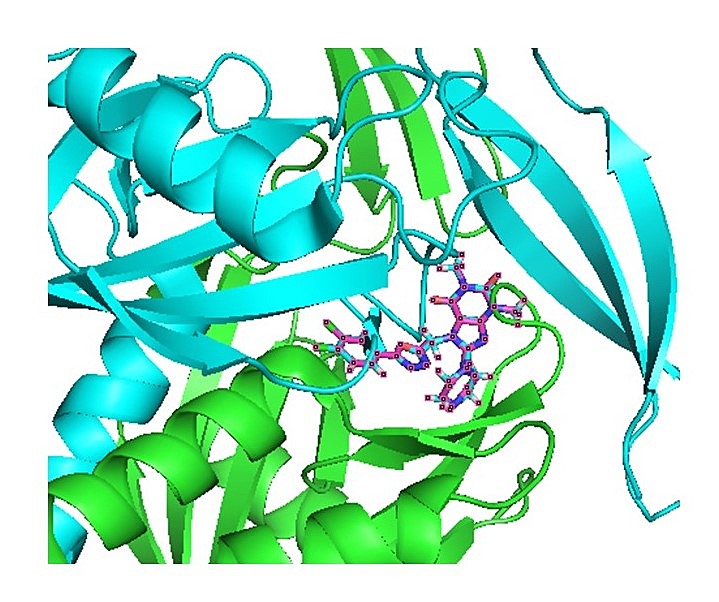
3D docking interaction analysis of ligand and target using PYMOL

The binding analysis (Table 2) indicates that these molecules can bind to the drug target efficiently and would be potential drugs for NUDT5. The negative and low value of ΔGbind indicates strong favorable bonds between protein and the ligand indicating that the ligand was in its most favorable conformations. The molecular docking was applied to explore the binding mechanism and studies on the novel ligand against the NUDT5 protein showed that the free binding energy for the inhibitor was small, indicating that the ligand binds favorably to the binding site. The ligand was observed as the best inhibitor candidate, which may be considered as a potential ligand for treatment of Breast cancer. The differences of binding affinities might be mainly attributed to the different hydrogen bond interactions and the orientation of ligands in the binding pocket. Therefore, this study thus helps to focus on in silico drug design for breast cancer based on its essential protein, NUDT5 identified as potential drug target. We show that the docked ligand binds at the same active site of the protein where the natural inhibitor (PDB ID: 5NWH) was bound (Figure 6)for further consideration and validation.

## Conclusion

Design and development of potential inhibitors to NUDT5 is of interest in the treatment of breast cancer. Hence, we report the structure based binding features of a lead compound 7-[[5-(3,4-dichlorophenyl)-1,3,4-oxadiazol-2-yl]methyl]-1,3-dimethyl-8-piperazin-1-yl-purine-2,6-dionewith NUDT5 for further in vitro and in vivo validation.

## Figures and Tables

**Table 1:** Properties of the control compound

Title	Formula	Weight	No. of Atoms
7-[[5-(3,4-dichlorophenyl)-1,3,4-oxadiazol-	C_20_H_20_Cl_2_N_8_O_3_	491.3306	33
2-yl]methyl]-1,3-dimethyl-8-piperazin-1-yl-purine-2,6-dione			
8-oxo-2'-deoxy-guanosine-5'-monophosphate	C_10_H_14_N_5_O_8_P	363.220621	24
Alpha-beta methylene ADP-ribose	C_16_H_25_N_5_O_13_P_2_	347.221221	23
Adenosine monophosphate	C_10_H_14_N_5_O_7_P	557.342922	36

**Table 2:** Ligand with its binding affinity and RMSD values from docking using PyRx

Ligand	Binding affinity (kcal/mol)	Mode	RMSD lower bound	RMSD upper bound
7-[[5-(3,4-dichlorophenyl)-1,3,4-oxadiazol-				
2-yl]methyl]-1,3-dimethyl-8-piperazin-1-yl-purine-2,6-dione	-9.2	0	0	0
8-oxo-2'-deoxy-guanosine-5'-monophosphate				
	-7.4	0	0	0
Alpha beta methylene ADP- ribose	-6.5	0	0	0
Adenosine monophosphate	-6.4	0	0	0

**Figure 4:**
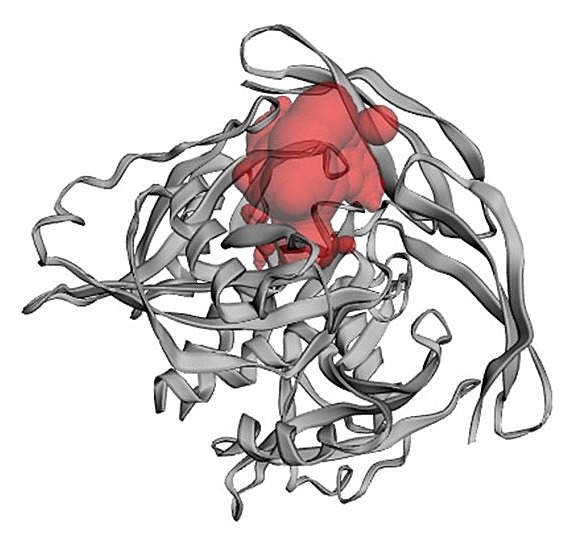
Binding site/active site analysis of NUDT5 using CASTp
